# Supplementary material for: Single-cell analysis of human glioma and immune cells identifies S100A4 as an immunotherapy target
Source: Nat Commun. 2022 Feb 9;13:767. doi: 10.1038/s41467-022-28372-y (PMC8828877; doi:10.1038/s41467-022-28372-y)
Supplement: Supplementary file 1 — Supplementary Information [file 41467_2022_28372_MOESM1_ESM.pdf]

a

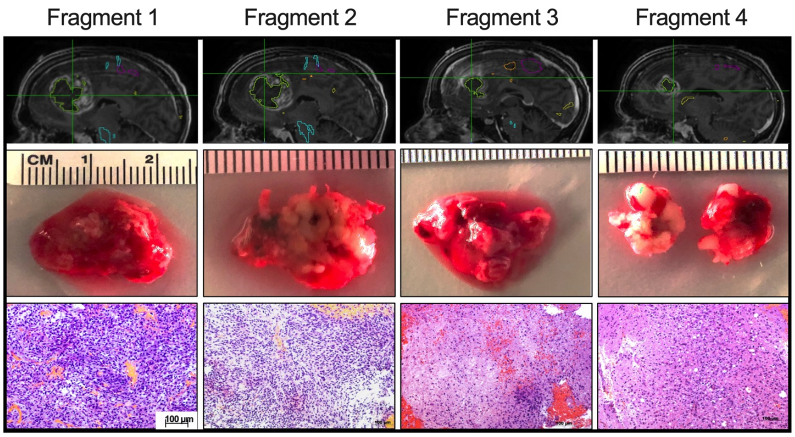

b

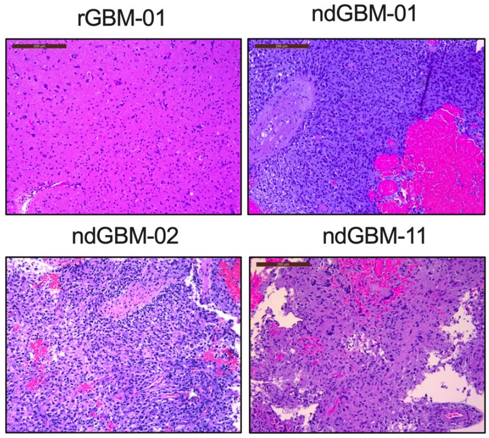

c

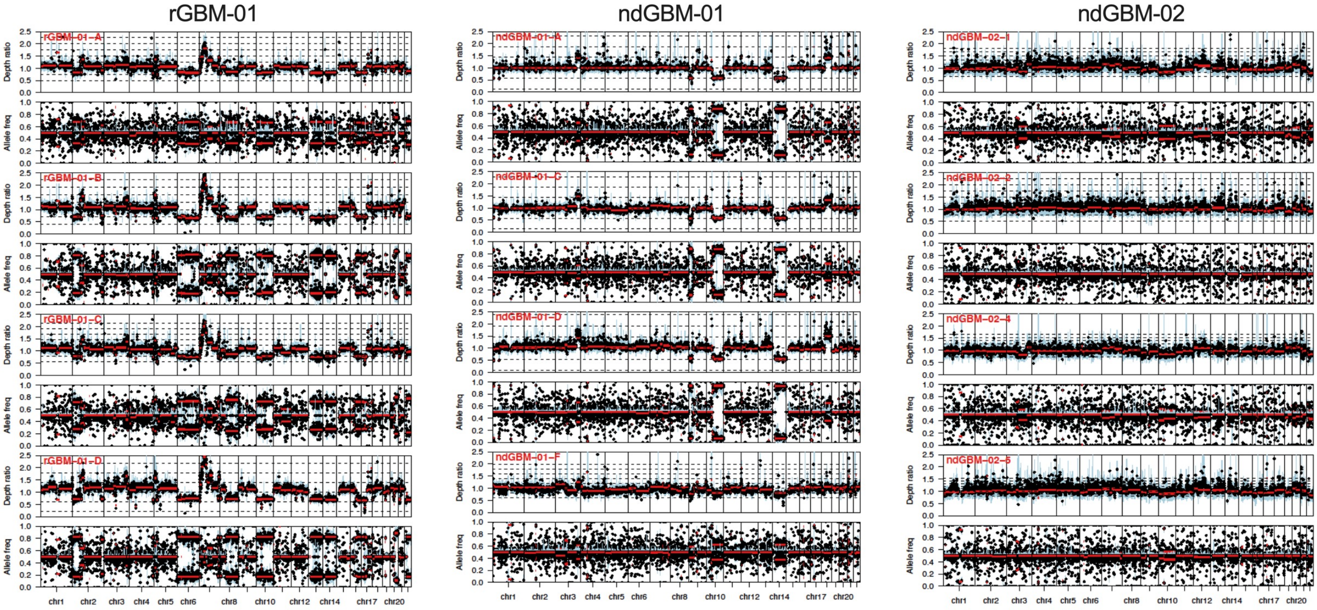

d

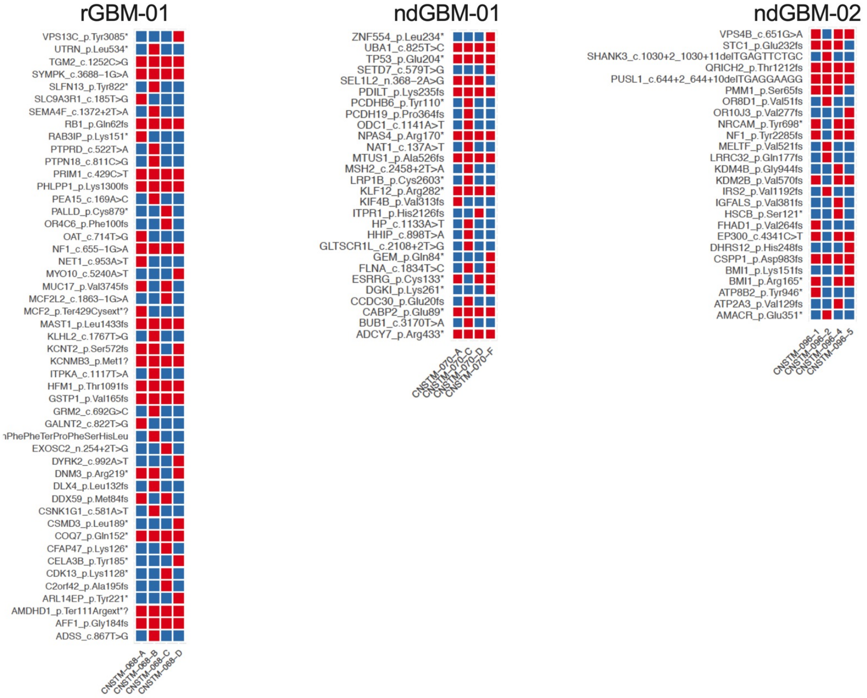

e

Fragment	#mutations
rGBM-01-A	24
rGBM-01-B	28
rGBM-01-C	22
rGBM-01-D	21
ndGBM-01-A	11
ndGBM-01-C	21
ndGBM-01-D	11
ndGBM-01-F	14
ndGBM-02-1	13
ndGBM-02-1	11
ndGBM-02-1	15
ndGBM-02-1	13

Supplementary Figure 1: Clinical and genomic characteristics of glioma patient samples analyzed. Multi-dimensional analysis was performed from 10 human glioma patients (3-4 fragments each). Multi-regional samples were collected from 4 primary GBM, 5 recurrent GBM and 1 low grade glioma patients. **a)** MRI images of sampling regions from ndGBM-2 patient, gross appearance and matching H&E staining of each fragment. **b)** H&E staining images showing the histology of four samples. Scale bar: 200 µm. **c)** Genome-wide plots indicating observed depth ratios and B-allele frequency (BAF) of genomic alterations identified in different regions of patient CNSTM-068, CNSTM-070, and 096. Red lines represent the segmented depth ratio or BAF values. **d)** High impact mutations identified per patient per fragment. For each sample, red block illustrates the presence and blue block represents the absence of identified mutation. **e)** Summary of number of high impact mutations per fragment.

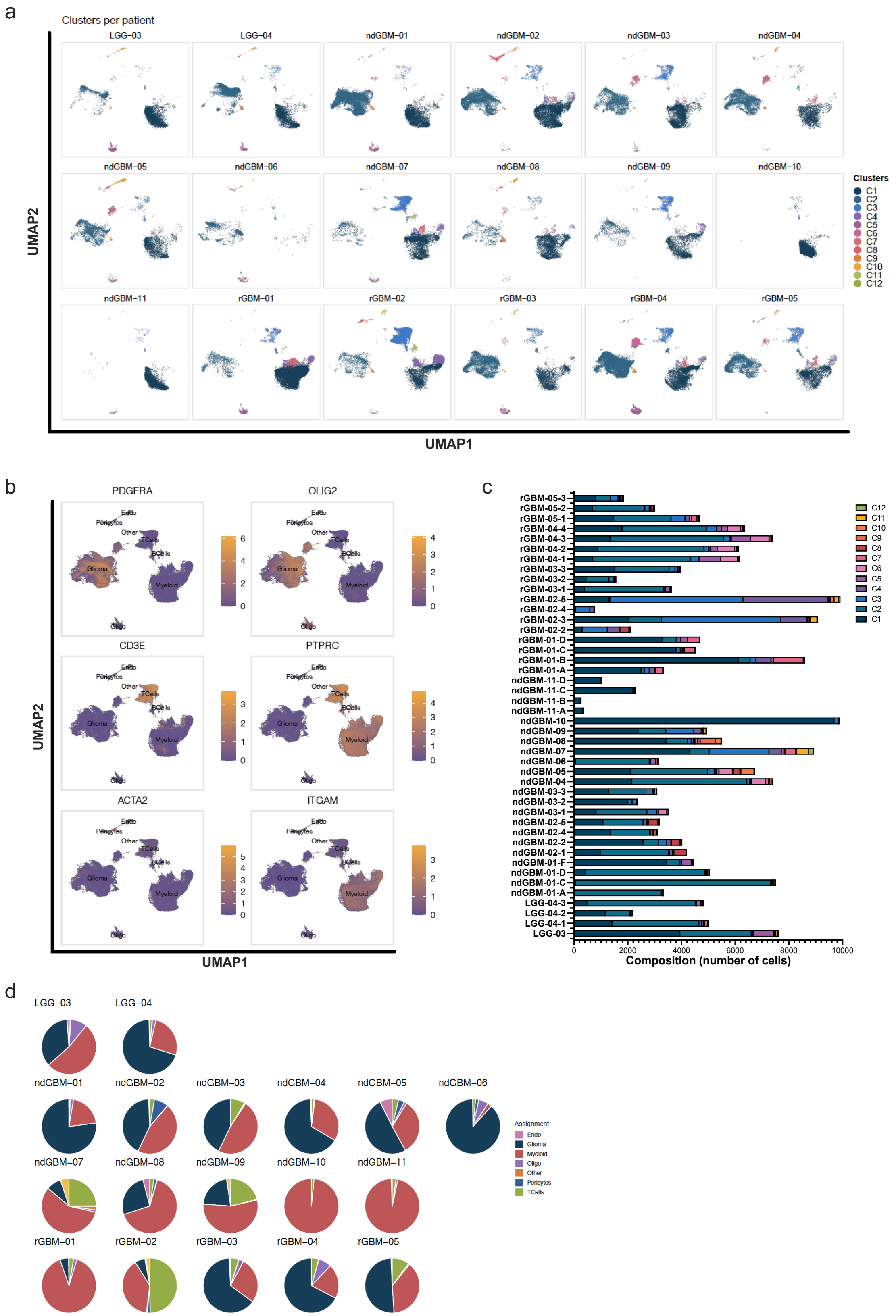

Supplementary Figure 2: Single cell RNA-seq data matrix for glioma patient samples analyzed. a) UMAP projections of identified clusters from 18 glioma patients, color-coded by cluster. **b)** Feature map showing expression of major lineage markers. **c)** Bar plots showing the distribution of cells in different clusters within each fragment from patients from whom multiple fragments were collected and analyzed separately. **d)** Pie charts representing percentages of cells per assignment by patient, color coded for cell type assignment. Source data for c and d are provided as a Source Data file.

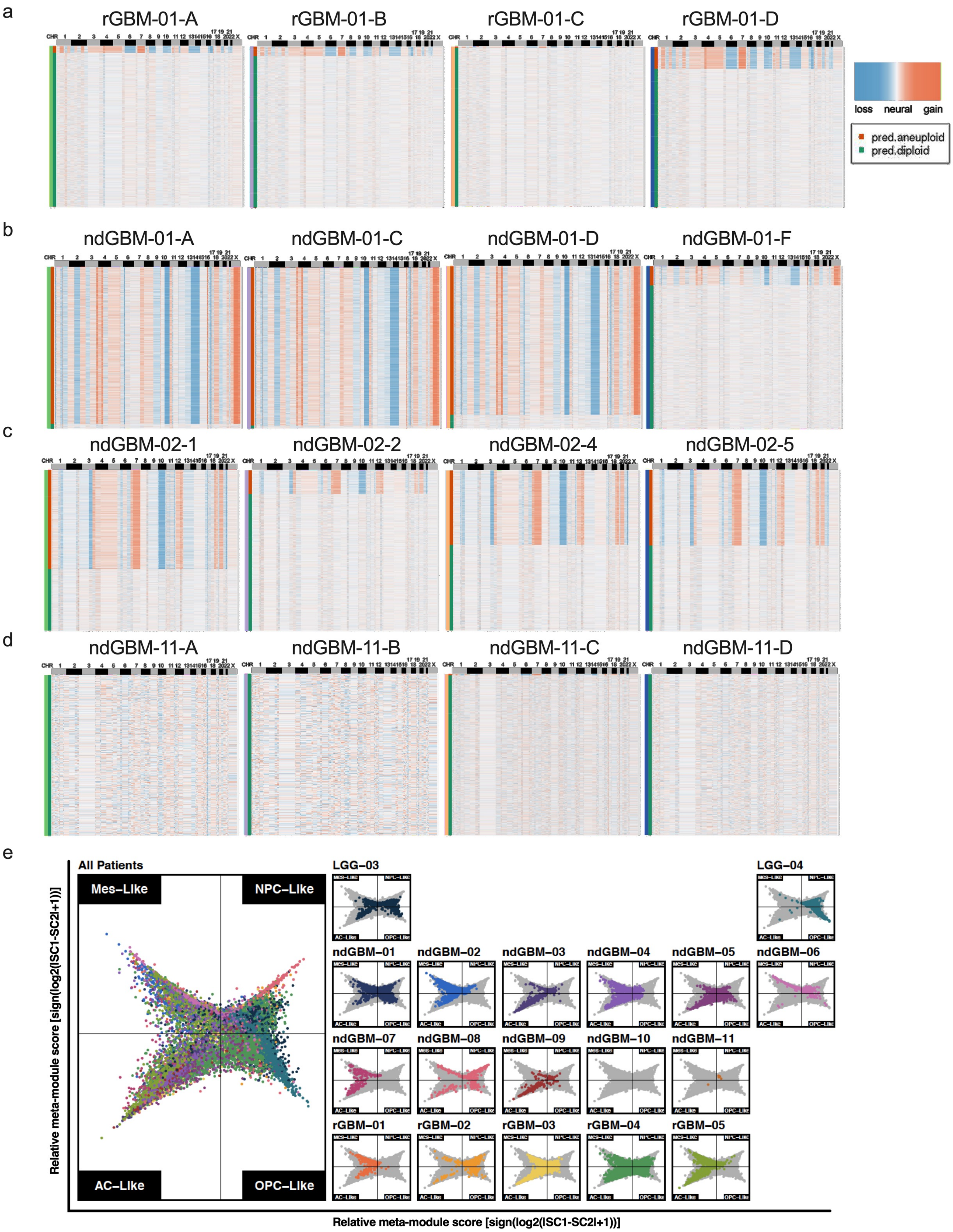

a
rGBM-01-A
rGBM-01-B
rGBM-01-C
rGBM-01-D
loss
neural
gain
pred.aneuploid
pred.diploid
b
ndGBM-01-A
ndGBM-01-C
ndGBM-01-D
ndGBM-01-F
c
ndGBM-02-1
ndGBM-02-2
ndGBM-02-4
ndGBM-02-5
d
ndGBM-11-A
ndGBM-11-B
ndGBM-11-C
ndGBM-11-D
e
All Patients
Mes–Like
NPC–Like
AC–Like
OPC–Like
LGG–03
LGG–04
ndGBM–01
ndGBM–02
ndGBM–03
ndGBM–04
ndGBM–05
ndGBM–06
ndGBM–07
ndGBM–08
ndGBM–09
ndGBM–10
ndGBM–11
rGBM–01
rGBM–02
rGBM–03
rGBM–04
rGBM–05
Relative meta-module score [sign(log2(|SC1-SC2|+1))]
Relative meta-module score [sign(log2(|SC1-SC2|+1))]

Supplementary Figure 3: Molecular characteristics of glioma cells. (a-d) Inferred copy number alterations in individual cells using the CopyKat algorithm: prediction of cancer and normal cells in each sample using single cell RNA-seq data. Copy number loss is marked in blue and gain is marked in red. Top is labeled with chromosome numbers. Each row represents a cell. Each panel is labeled with patient ID and fragment number. **(e)** Two-dimensional butterfly representation of molecular subtype signature scores per Neftel *et al*. Each quadrant corresponds to one subtype (mesenchymal like (Mes-like), NPC-like, AC-like and OPC-like), and the exact position of each cell reflects its relative signature scores. See Methods for details.

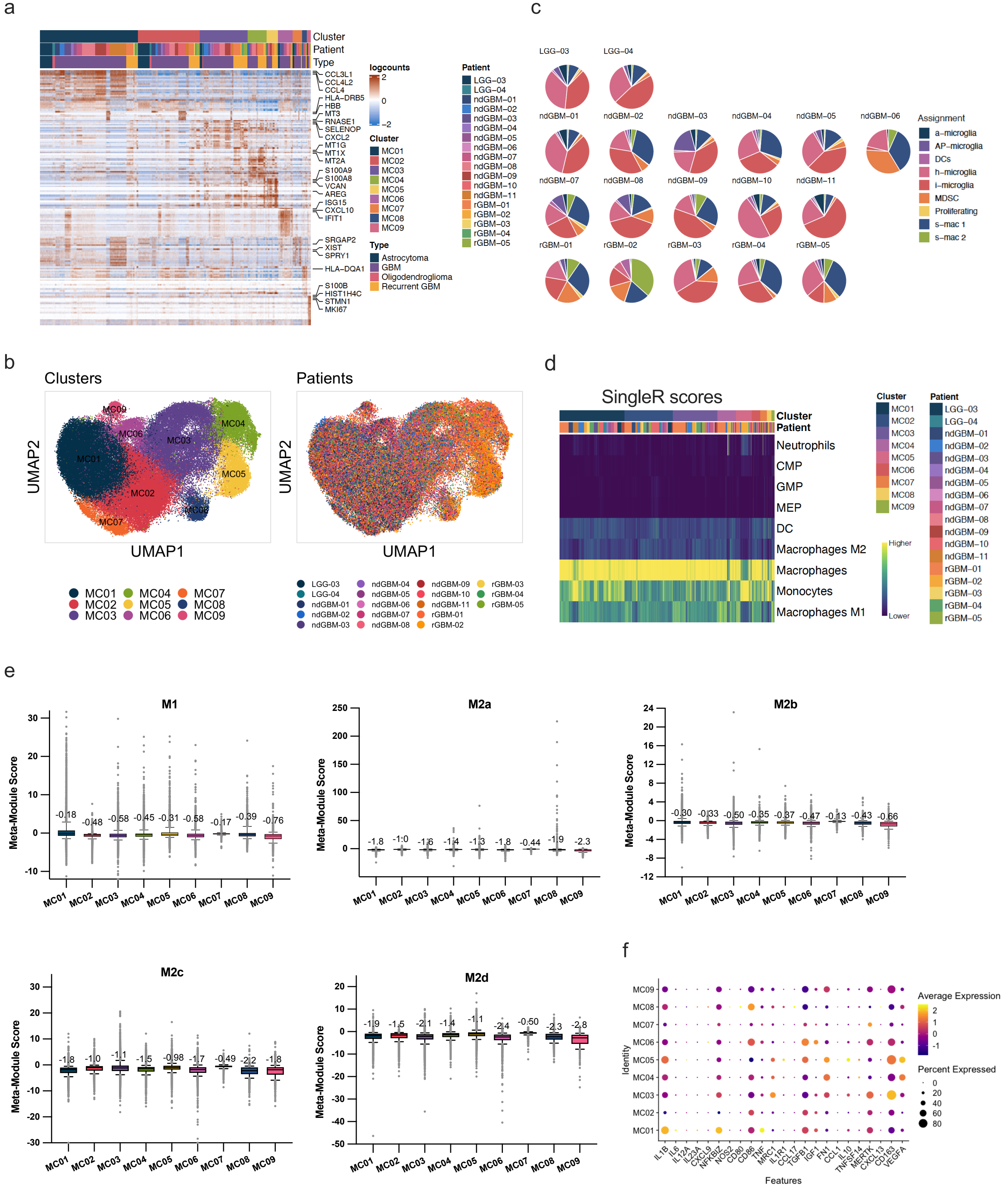

a
Cluster
Patient
Type
CCL3L1
CCL4L2
CCL4
HLA-DRB5
HBB
MT3
RNASE1
SELENOP
CXCL2
MT1G
MT1X
MT2A
S100A9
S100A8
VCAN
AREG
ISG15
CXCL10
IFIT1
SRGAP2
XIST
SPRY1
HLA-DQA1
S100B
HIST1H4C
STMN1
MKI67
logcounts
2
0
−2
Cluster
MC01
MC02
MC03
MC04
MC05
MC06
MC07
MC08
MC09
Type
Astrocytoma
GBM
Oligodendroglioma
Recurrent GBM
Patient
LGG−03
LGG−04
ndGBM−01
ndGBM−02
ndGBM−03
ndGBM−04
ndGBM−05
ndGBM−06
ndGBM−07
ndGBM−08
ndGBM−09
ndGBM−10
ndGBM−11
rGBM−01
rGBM−02
rGBM−03
rGBM−04
rGBM−05
c
LGG−03
LGG−04
ndGBM−01
ndGBM−02
ndGBM−03
ndGBM−04
ndGBM−05
ndGBM−06
ndGBM−07
ndGBM−08
ndGBM−09
ndGBM−10
ndGBM−11
rGBM−01
rGBM−02
rGBM−03
rGBM−04
rGBM−05
Assignment
a−microglia
AP−microglia
DCs
h−microglia
i−microglia
MDSC
Proliferating
s−mac 1
s−mac 2
b
Clusters
Patients
UMAP2
UMAP1
MC01
MC02
MC03
MC04
MC05
MC06
MC07
MC08
MC09
LGG-03
LGG-04
ndGBM-01
ndGBM-02
ndGBM-03
ndGBM-04
ndGBM-05
ndGBM-06
ndGBM-07
ndGBM-08
ndGBM-09
ndGBM-10
ndGBM-11
rGBM-01
rGBM-02
rGBM-03
rGBM-04
rGBM-05
d
SingleR scores
Cluster
Patient
Neutrophils
CMP
GMP
MEP
DC
Macrophages M2
Macrophages
Monocytes
Macrophages M1
Higher
Lower
e
M1
M2a
M2b
M2c
M2d
Meta-Module Score
MC01
MC02
MC03
MC04
MC05
MC06
MC07
MC08
MC09
-0.18
-0.48
-0.58
-0.45
-0.31
-0.58
-0.17
-0.39
-0.76
-1.8
-1.0
-1.6
-1.4
-1.3
-1.8
-0.44
-1.9
-2.3
-0.30
-0.33
-0.50
-0.35
-0.37
-0.47
-0.13
-0.43
-0.66
-1.8
-1.0
-1.1
-1.5
-0.98
-1.7
-0.49
-2.2
-1.8
-1.9
-1.5
-2.1
-1.4
-1.1
-2.4
-0.50
-2.3
-2.8
f
Identity
Features
IL1B
IL6
IL12A
IL23A
CXCL9
NFKBIZ
NOS2
CD80
CD86
TNF
MRC1
IL1R1
CCL17
TGFB1
IGF1
FN1
CCL1
IL10
TNFSF14
MERTK
CXCL13
CD163
VEGFA
Average Expression
2
1
0
-1
Percent Expressed
0
20
40
60
80

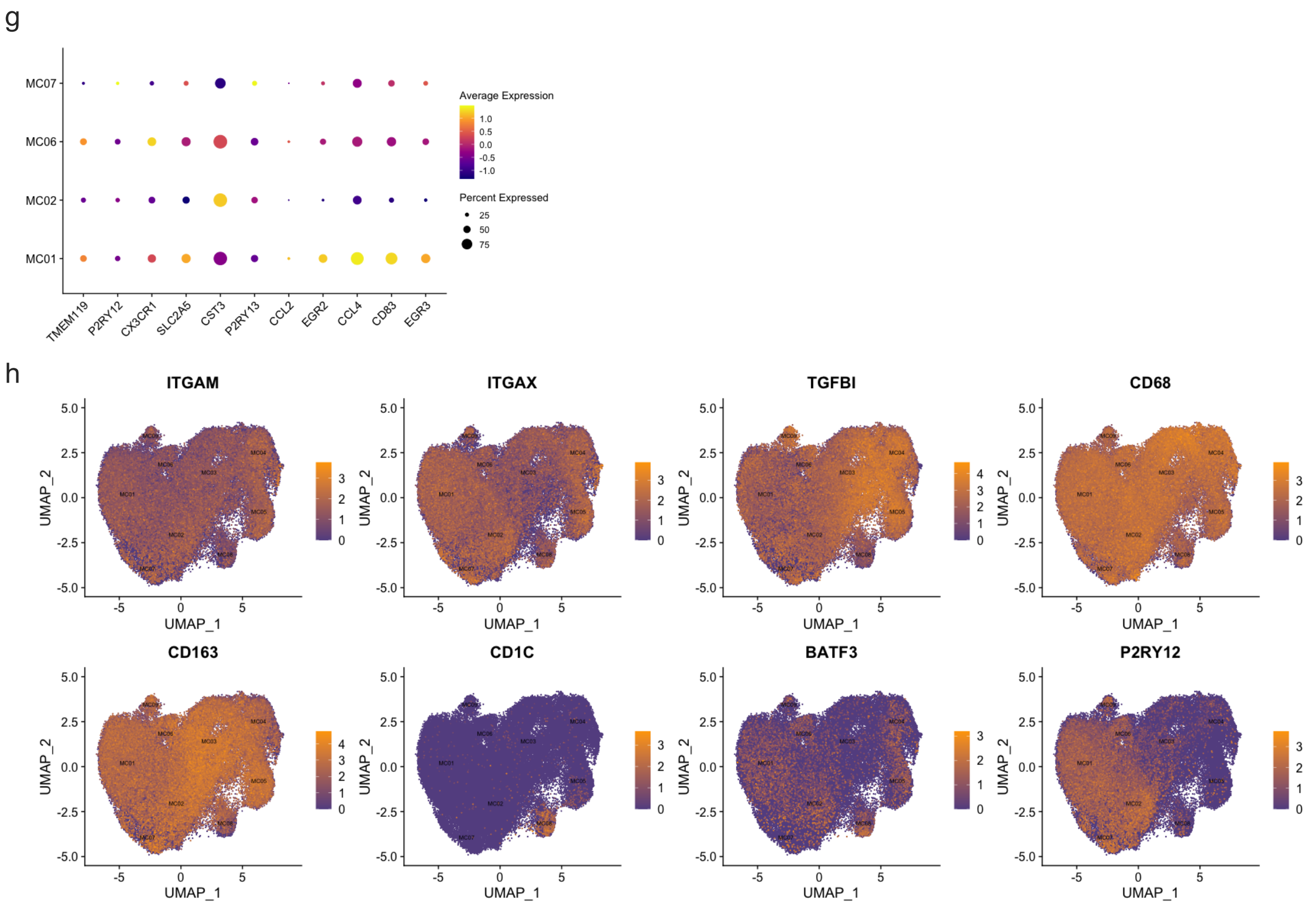

Supplementary Figure 4: De novo clustering of glioma associated macrophages identify nine subtypes that cannot be distinguished by *in vitro* defined M0, M1, and M2-macrophage marker genes. a) 83,479 cells from clusters 1, 4, and 7 in **Figure 1** corresponding to myeloid cells were extracted and used for *de novo* clustering. Nine myeloid clusters (MCs) are identified, and the top 20 differentially expressed genes, ranked by FDR, are shown in the heatmap. **b)** UMAP projections showing contribution of 18 patient samples and different clusters. **c)** Pie charts representing percentage of cells, per assignment by patient, color coded for cell type assignment. **d)** Heatmap of SingleR cell-type annotation scores, derived by comparing single-cell expression profiles against inbuilt blueprint_encode reference database from SingleR package, yellow and green colors show higher and lower scores respectively. **e)** Box plot representing the signature scores of *in vitro* defined macrophage in our glioma associated myeloid clusters. Lines in the middle represent median. Mean values are marked inside each box plot. Whiskers represent 10th and 90th values, the line inside the box represents the mean and the box extends from the 25th to 75th percentiles. Median values are indicated on top of each box. Scores were calculated using counts from a random subset of 52,112 myeloid cells (MC01 n=10,000, MC02 n=10,000, MC03 n=10,000, MC04 n=7,206, MC05 n=6,142, MC06 n=3,303, MC07 n=2,594, MC08 n=1,715, MC09 n=1,152). **f)** Dot plots of in vitro defined macrophage (M0/M1/M2) marker genes. Average expression of highlighted genes across all myeloid clusters. The size of the dot shows the percentage of cells expressing a particular gene while color shows the average gene expression levels. (navy is low and yellow is high). **g)** Dot plot showing markers for homeostatic and activated microglia. **h)** Feature plots of select microglia, macrophage and DC markers. Source data for c and e are provided as a Source Data file.

a

b

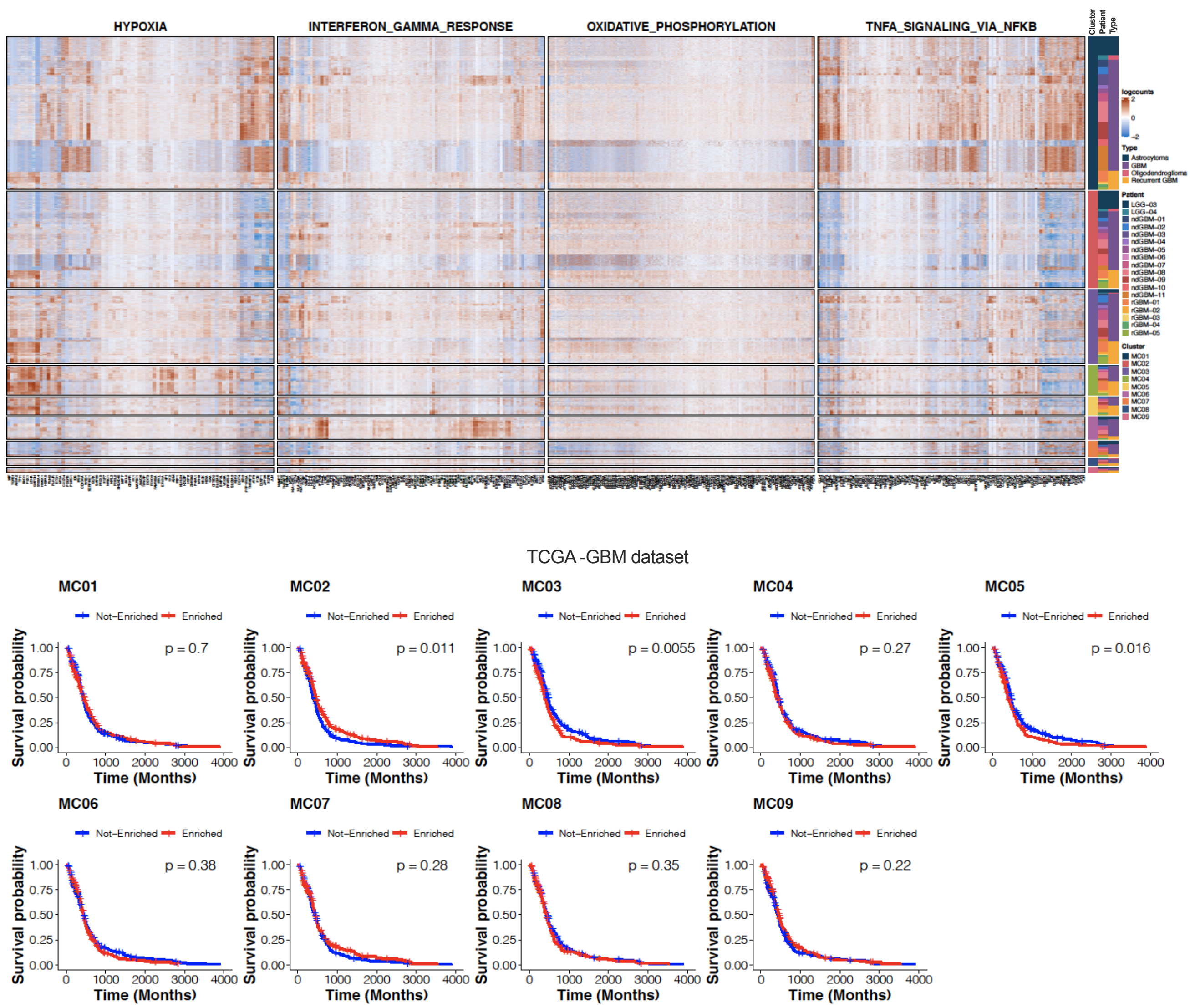

Supplementary Figure 5: Individual myeloid clusters show enrichment of overlapping but unique hallmark pathway activation. a) Heatmaps showing expression of select GSEA Hallmark Pathway signature genes in myeloid cells. Gene expression values were centered, scaled, and transformed to a scale from -2 to 2. **b)** Kaplan-Meier survival curves representing the survival of TCGA GBM patients(n=528), stratified by positive (Enriched), or negative (Not Enriched) signature scores for each the myeloid clusters. Zero cell score values were used as cutoffs for positive or negative designations. *P*-values represent the Log-rank Mantel Cox test. Source data for b are provided as a Source Data file.

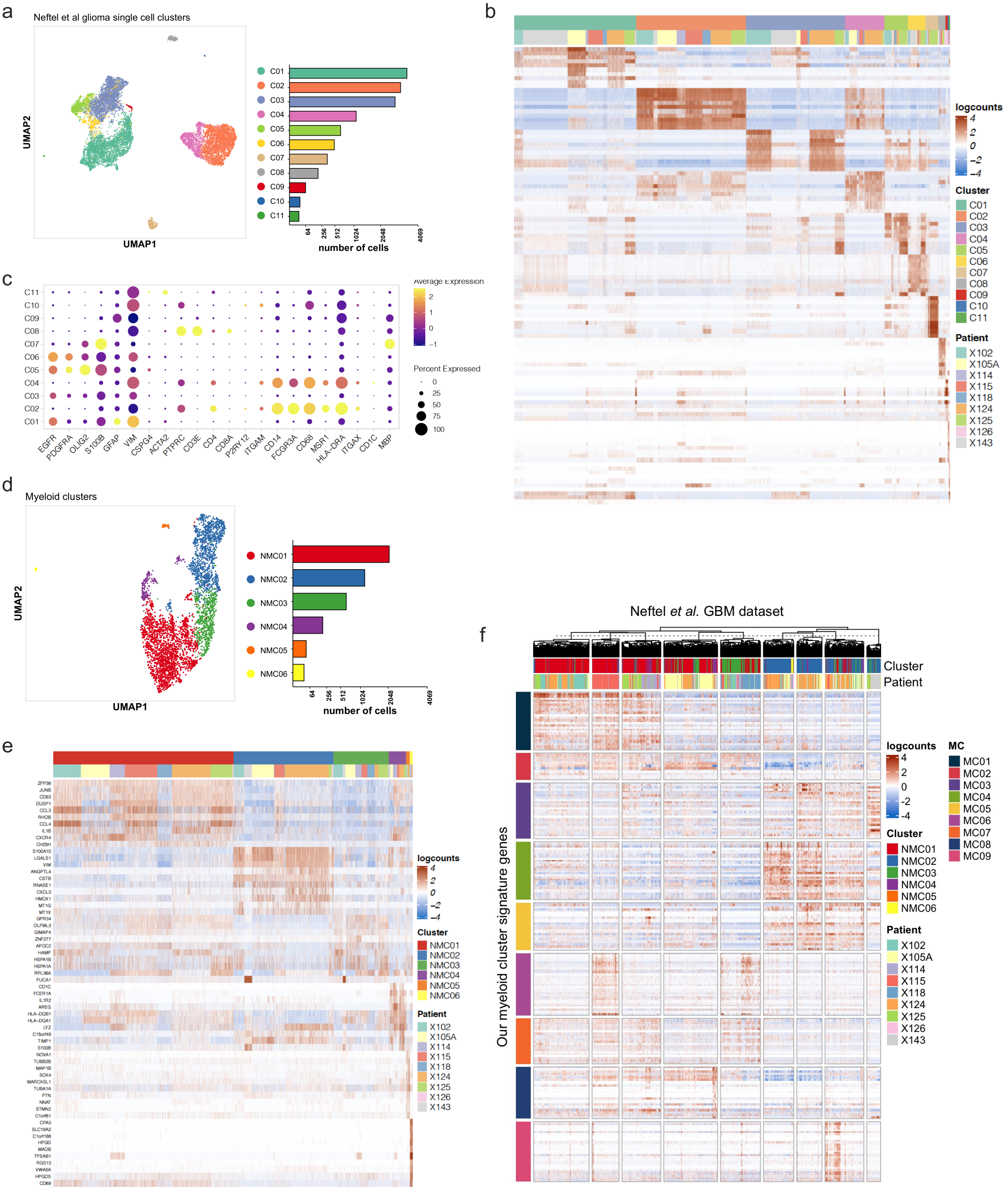
a
Neftel et al glioma single cell clusters
UMAP2
UMAP1
C01
C02
C03
C04
C05
C06
C07
C08
C09
C10
C11
64
256
512
1024
2048
4069
number of cells
b
logcounts
4
2
0
−2
−4
Cluster
C01
C02
C03
C04
C05
C06
C07
C08
C09
C10
C11
Patient
X102
X105A
X114
X115
X118
X124
X125
X126
X143
c
C11
C10
C09
C08
C07
C06
C05
C04
C03
C02
C01
EGFR
PDGFRA
OLIG2
S100B
GFAP
VIM
CSPG4
ACTA2
PTPRC
CD3E
CD4
CD8A
P2RY12
ITGAM
CD14
FCGR3A
CD68
MSR1
HLA-DRA
ITGAX
CD1C
MBP
Average Expression
2
1
0
−1
Percent Expressed
0
25
50
75
100
d
Myeloid clusters
UMAP2
UMAP1
NMC01
NMC02
NMC03
NMC04
NMC05
NMC06
64
256
512
1024
2048
4069
number of cells
e
logcounts
4
2
0
−2
−4
Cluster
NMC01
NMC02
NMC03
NMC04
NMC05
NMC06
Patient
X102
X105A
X114
X115
X118
X124
X125
X126
X143
f
Neftel *et al.* GBM dataset
Cluster
Patient
Our myeloid cluster signature genes
logcounts
4
2
0
-2
-4
Cluster
NMC01
NMC02
NMC03
NMC04
NMC05
NMC06
Patient
X102
X105A
X114
X115
X118
X124
X125
X126
X143
MC
MC01
MC02
MC03
MC04
MC05
MC06
MC07
MC08
MC09

Supplementary Figure 6: Independent GBM cohort validation. Single cell RNA sequencing data from the Neftel *et al.,* (#GSE131928, GEO) were analyzed as an independent dataset. **a) A** UMAP projection of 16,201 single cells color-coded by identified clusters(left). Bar plots show number of cells per cluster (right). **b)** A heatmap showing expression of top 10 differentially expressed genes, ranked by FDR, in each cluster. Gene expression values were centered, scaled, and transformed to a scale from -4 to 4. **c)** A dot plot showing the average expression of lineage markers across all cells within each cluster. The size of the dot shows the percentage of cells expressing a particular gene while color shows the average gene expression levels (navy is low and yellow is high). Clusters 2 and 4 were identified as myeloid cells. **d**) A UMAP projection of de novo clustered 5,739 myeloid single cells (C02 and C04 in **a)**), color-coded by identified clusters(left). Bar plots show number of cells per myeloid cluster (right). **e)** A heatmap showing expression of top 10 differentially expressed genes, ranked by FDR, in each cluster. Gene expression values were centered, scaled, and transformed to a scale from -4 to 4. **f)** Unsupervised hierarchical clustering with our myeloid cluster signature genes (Supplementary Table 9) segregates anti-tumorigenic microglia to the left branch (enriched in MC1, MC2, MC7 genes) and pro-tumorigenic BMDMs to the right branch (enriched with MC3, MC4, MC5 genes). Rows represent our myeloid cluster signature genes and columns represent individual myeloid cells from the Neftel *et al.,* dataset. Gene expression values were centered, scaled, and transformed to a scale from -4 to 4. Source data for a and d are provided as a Source Data file.

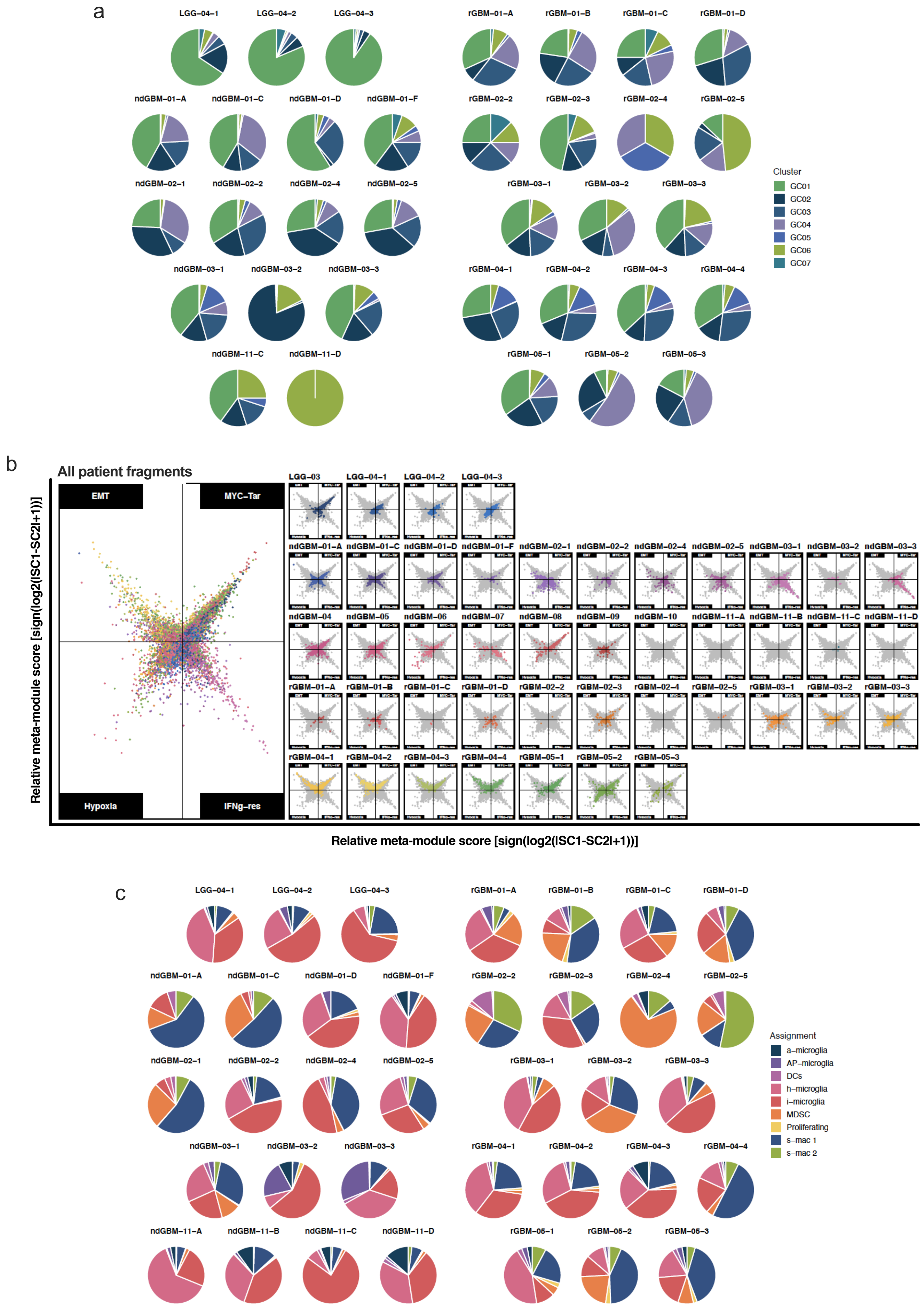
a
LGG–04–1
LGG–04–2
LGG–04–3
rGBM–01–A
rGBM–01–B
rGBM–01–C
rGBM–01–D
ndGBM–01–A
ndGBM–01–C
ndGBM–01–D
ndGBM–01–F
rGBM–02–2
rGBM–02–3
rGBM–02–4
rGBM–02–5
ndGBM–02–1
ndGBM–02–2
ndGBM–02–4
ndGBM–02–5
rGBM–03–1
rGBM–03–2
rGBM–03–3
ndGBM–03–1
ndGBM–03–2
ndGBM–03–3
rGBM–04–1
rGBM–04–2
rGBM–04–3
rGBM–04–4
ndGBM–11–C
ndGBM–11–D
rGBM–05–1
rGBM–05–2
rGBM–05–3
Cluster
GC01
GC02
GC03
GC04
GC05
GC06
GC07
b
All patient fragments
EMT
MYC–Tar
Hypoxia
IFNg–res
LGG–03
LGG–04–1
LGG–04–2
LGG–04–3
ndGBM–01–A
ndGBM–01–C
ndGBM–01–D
ndGBM–01–F
ndGBM–02–1
ndGBM–02–2
ndGBM–02–4
ndGBM–02–5
ndGBM–03–1
ndGBM–03–2
ndGBM–03–3
ndGBM–04
ndGBM–05
ndGBM–06
ndGBM–07
ndGBM–08
ndGBM–09
ndGBM–10
ndGBM–11–A
ndGBM–11–B
ndGBM–11–C
ndGBM–11–D
rGBM–01–A
rGBM–01–B
rGBM–01–C
rGBM–01–D
rGBM–02–2
rGBM–02–3
rGBM–02–4
rGBM–02–5
rGBM–03–1
rGBM–03–2
rGBM–03–3
rGBM–04–1
rGBM–04–2
rGBM–04–3
rGBM–04–4
rGBM–05–1
rGBM–05–2
rGBM–05–3
Relative meta-module score [sign(log2(|SC1-SC2|+1))]
Relative meta-module score [sign(log2(|SC1-SC2|+1))]
c
LGG–04–1
LGG–04–2
LGG–04–3
rGBM–01–A
rGBM–01–B
rGBM–01–C
rGBM–01–D
ndGBM–01–A
ndGBM–01–C
ndGBM–01–D
ndGBM–01–F
rGBM–02–2
rGBM–02–3
rGBM–02–4
rGBM–02–5
ndGBM–02–1
ndGBM–02–2
ndGBM–02–4
ndGBM–02–5
rGBM–03–1
rGBM–03–2
rGBM–03–3
ndGBM–03–1
ndGBM–03–2
ndGBM–03–3
rGBM–04–1
rGBM–04–2
rGBM–04–3
rGBM–04–4
ndGBM–11–A
ndGBM–11–B
ndGBM–11–C
ndGBM–11–D
rGBM–05–1
rGBM–05–2
rGBM–05–3
Assignment
a–microglia
AP–microglia
DCs
h–microglia
i–microglia
MDSC
Proliferating
s–mac 1
s–mac 2

d

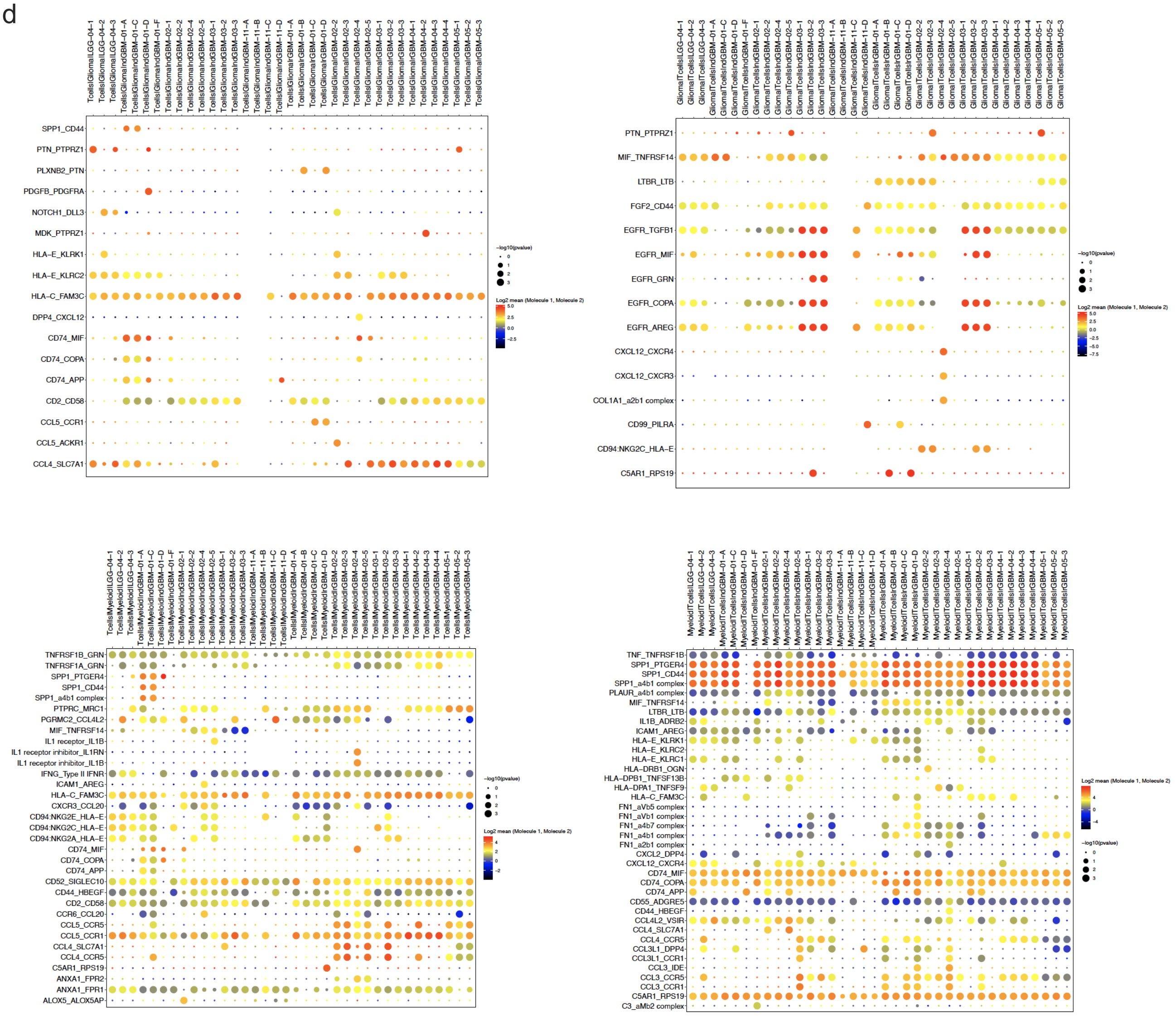

Supplementary Figure 7: Spatial heterogeneity in glioma, T cell and myeloid subsets. a) Pie charts representing percentage of glioma cells per cluster by fragment, color coded by cell subtype clusters. **b)** Two-dimensional butterfly plot visualization of top Hallmark pathways from GSEA (EPITHELIAL_MESENCHYMAL_TRANSITION, MYC_TARGETS_V1, HYPOXIA, and INTERFERON_GAMMA_RESPONSE) in different fragments, representing signature scores as relative meta-module scores. Colors represent different clusters. **c)** Pie charts representing percentage of myeloid cells by fragment, color coded for cell type assignment. **d)** Cell:cell communication analysis using CellphoneDB. Dot plots showing ligand:receptor interactions between glioma:T-cells , T-cells:glioma and T-cells:myeloid cells, and myeloid:T-cells in different fragments. Each dot size shows the -log10 *p*-value and color indicates the log2 mean of expression values for the listed LR pairs (Y-axis) in the respective interacting cell types (X-axis, top). Only significant LR pairs, with cutoffs of *p*-value <= 0.05 and log2 mean expression value >2, are shown. The *p*-values were generated by CellphoneDB which uses a one-sided permutation-test to compute significant interactions. Source data for a and c are provided as a Source Data file.

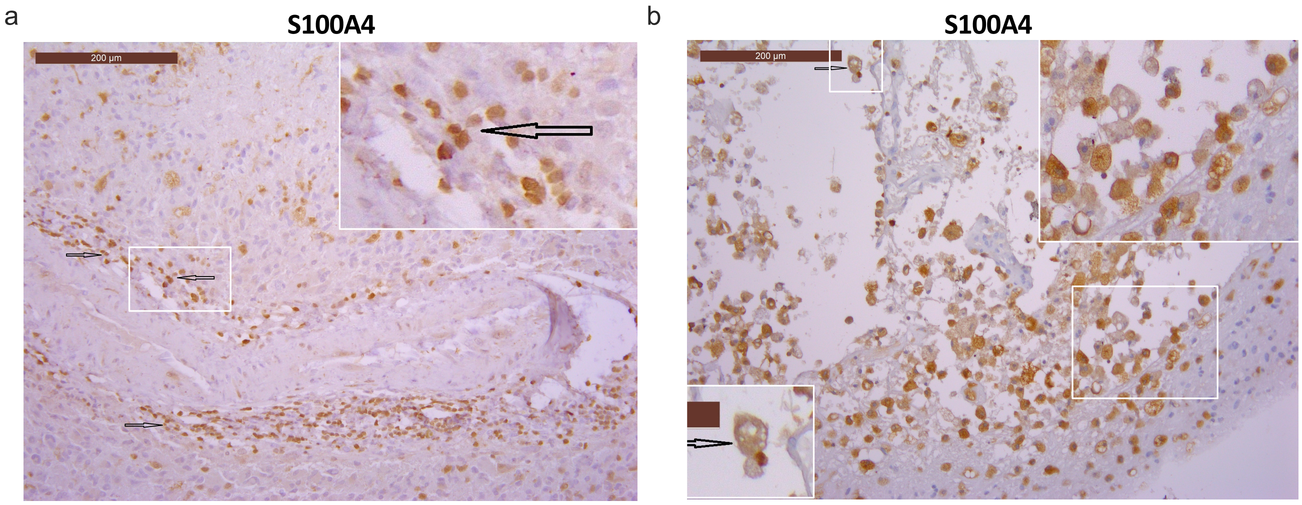

Supplementary Figure 8: S100A4 staining in human GBM. (a) Intermediate magnification of a glioma showing perivascular lymphocytic infiltrates with hemosiderin deposition. (Lymphocytes highlighted at arrows) The corresponding S100A4 immunostain highlights the immunoreactivity of the perivascular lymphocytes as well as of scattered neoplastic cells. Scale bar = 200 µm. Image is representative of 22 patient samples stained. **b)** S100A4 staining showing numerous foamy macrophages infiltrating the tissue and filling the cavitated spaces. The arrow highlights an individual macrophage. Scale bar = 200 µm . Image is representative of 22 patient samples stained.

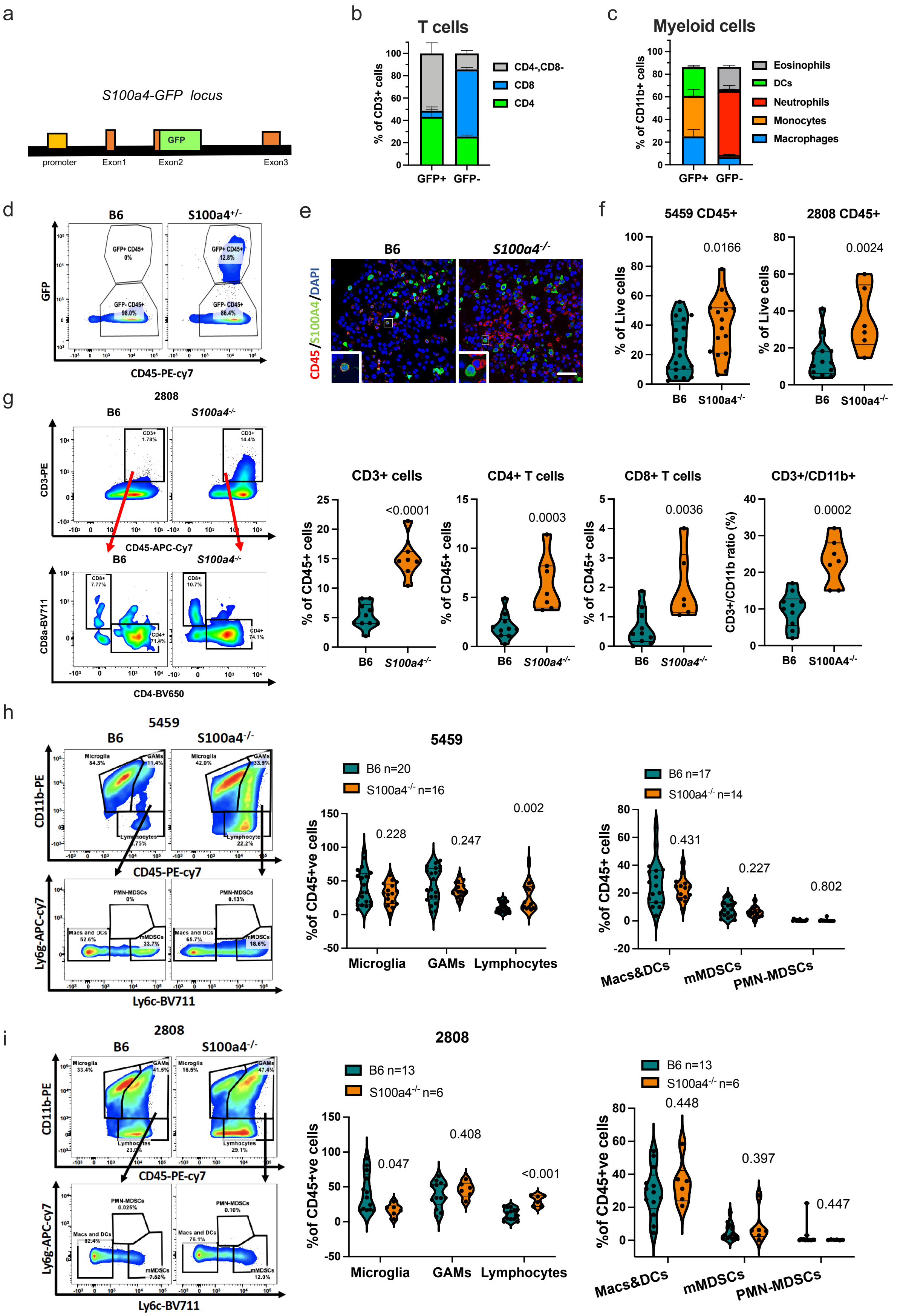

a
S100a4-GFP locus
promoter
Exon1
Exon2
Exon3
GFP
b
T cells
% of CD3+ cells
CD4-,CD8-
CD8
CD4
GFP+ GFP-
c
Myeloid cells
% of CD11b+ cells
Eosinophils
DCs
Neutrophils
Monocytes
Macrophages
GFP+ GFP-
d
B6
S100a4+/-
GFP
CD45-PE-cy7
e
B6
S100a4-/-
CD45/S100A4/DAPI
f
5459 CD45+
2808 CD45+
% of Live cells
0.0166
0.0024
B6 S100a4-/-
g
2808
CD3-PE
CD45-APC-Cy7
CD8a-BV711
CD4-BV650
CD3+ cells
CD4+ T cells
CD8+ T cells
CD3+/CD11b+
% of CD45+ cells
CD3+/CD11b ratio (%)
<0.0001
0.0003
0.0036
0.0002
h
5459
CD11b-PE
Ly6g-APC-cy7
Ly6c-BV711
B6 n=20
S100a4-/- n=16
B6 n=17
S100a4-/- n=14
%of CD45+ve cells
%of CD45+ cells
0.228
0.247
0.002
0.431
0.227
0.802
Microglia GAMs Lymphocytes
Macs&DCs mMDSCs PMN-MDSCs
i
2808
B6 n=13
S100a4-/- n=6
B6 n=13
S100a4-/- n=6
0.047
0.408
<0.001
0.448
0.397
0.447
Microglia GAMs Lymphocytes
Macs&DCs mMDSCs PMN-MDSCs

Supplementary Figure 9: Deletion of *S100a4* in host microenvironment reprograms the GBM immune landscape and confers significant survival advantage. a) A schematic of *S100a4*$^{-/-}$ mouse locus, where GFP cDNA replaces exon2 of the *S100a4* gene. In this strain, *S100a4*-expresing cells can be identified by the GFP reporter expression in heterozygous and homozygous mice. **b)** Stacked bar graphs representing T cell populations withing GFP+ and GFP- PBMCs. **c)** Stacked bar graphs representing myeloid cell populations within GFP+ and GFP- PBMCs. N=4. **d)** Dot plots showing GFP^{+ve} population in *S100a4*$^{+/-}$ blood . **e)** Immunofluorescence analysis showing the co-expression of S100A4 with CD45+ cells in B6 wildtype but not *S100a4*$^{-/-}$ tumors. n=3 each. Scale bar: 50 µm. **f)** Violin plots showing the percentage of CD45+ glioma associated immune cells in B6 vs *S100a4*$^{-/-}$ host mice intracranially injected with either 5459 (left B6 n=20, *S100a4*$^{-/-}$ n=16), or 2808 (right B6 n=13, *S100a4*$^{-/-}$ n=6) S100ß-vERBb;p53 mouse gliomas. All pairwise analyses were performed using two-tailed T-tests**. g)** Flow cytometric analysis of tumor-infiltrating T-cells in B6 vs *S100a4*$^{-/-}$ host mice intracranially injected with 2808 primary mouse gliomas (B6=10, *S100a4*$^{-/-}$ =7) . All pairwise analyses were performed using two-tailed t-tests. **h-i)** Flow cytometric analysis of tumor-infiltrating myeloid cells in B6 vs *S100a4*$^{-/-}$ host mice intracranially injected with either 5459 (h-N B6 n=20, *S100a4*$^{-/-}$ *n*=16), or 2808 (i-B6 n=12*, S100a4*$^{-/-}$ n=7) primary mouse glioma transplant. All pairwise analyses were performed using two-tailed t-tests. Source data for b, c, f, g, h and i are provided as a Source Data file.

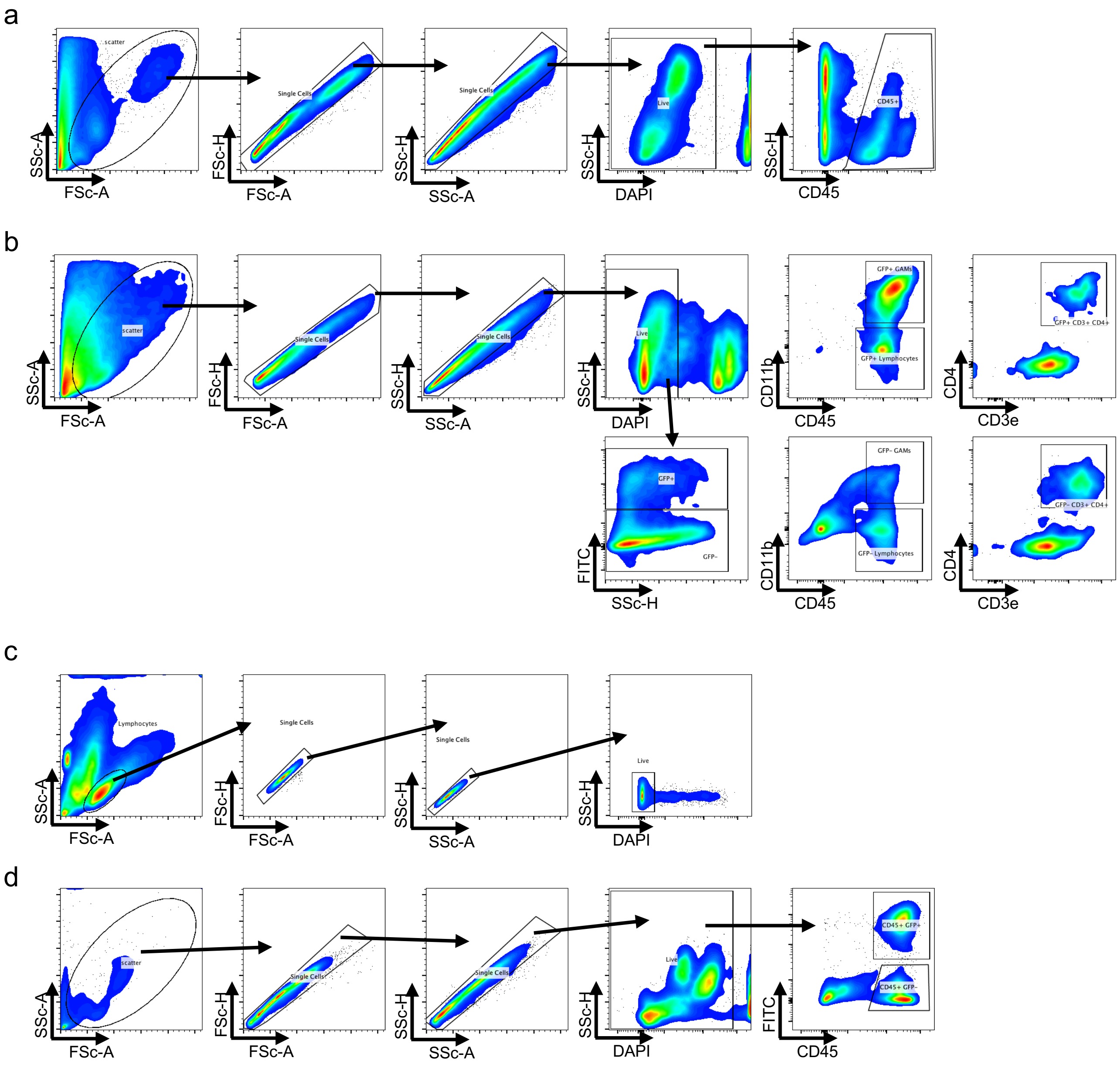

Supplementary Figure 10: Gating Strategy. a) Gating strategy for CD45+ cells from mouse gliomas presented on Fig. 6h,I and Supp Fig 9f, g, h and i. **b)** Gating strategy to sort GFP+/- GAMs and T cells from mouse gliomas presented on Fig. 7. **c)** Gating strategy for T cell proliferation assay presented on Fig. 7e. **d)** Gating strategy for GFP+/- CD45+ cells from mouse blood presented on Supp Fig b,c and d.
