# Supplementary material for: Single-cell analysis of human glioma and immune cells identifies S100A4 as an immunotherapy target
Source: Nat Commun. 2022 Feb 9;13:767. doi: 10.1038/s41467-022-28372-y (PMC8828877; doi:10.1038/s41467-022-28372-y)
Supplement: Supplementary file 3 — Description of Additional Supplementary Files [file 41467_2022_28372_MOESM3_ESM.pdf]

Abdelfattah et al: Description of Additional Supplementary Files

File name: Supplementary Data1
Description: Clinical information of the glioma patients.

File name: Supplementary Data2
Description: Number of cells in all clusters per patient and category

File name: Supplementary Data3
Description: Differentially expressed genes in all clusters

File name: Supplementary Data4
Description: Number of cells in glioma clusters per patient and category

File name: Supplementary Data5
Description: Differentially expressed genes in glioma clusters

File name: Supplementary Data6
Description: Number of cells in T cells clusters per patient and category

File name: Supplementary Data7
Description: Differentially expressed genes in T cell clusters

File name: Supplementary Data8
Description: Number of cells in myeloid clusters per patient and category

File name: Supplementary Data9
Description: Differentially expressed genes in Myeloid clusters

File name: Supplementary Data10
Description: Multivariate analysis summary

File name: Supplementary Data11
Description: Number of cells per fragment
