# Supplementary material for: Single-cell analysis of human glioma and immune cells identifies S100A4 as an immunotherapy target
Source: Nat Commun. 2022 Feb 9;13:767. doi: 10.1038/s41467-022-28372-y (PMC8828877; doi:10.1038/s41467-022-28372-y)
Supplement: Supplementary file 15 — Reporting summary [file 41467_2022_28372_MOESM15_ESM.pdf]

nature research

Corresponding author(s): Kyuson Yun

Last updated by author(s): 12-13-2021

Reporting Summary

Nature Research wishes to improve the reproducibility of the work that we publish. This form provides structure for consistency and transparency in reporting. For further information on Nature Research policies, see our Editorial Policies and the Editorial Policy Checklist.

Statistics

For all statistical analyses, confirm that the following items are present in the figure legend, table legend, main text, or Methods section.

n/a	Confirmed	
[ ]	[x]	The exact sample size (*n*) for each experimental group/condition, given as a discrete number and unit of measurement
[x]	[ ]	A statement on whether measurements were taken from distinct samples or whether the same sample was measured repeatedly
[ ]	[x]	The statistical test(s) used AND whether they are one- or two-sided<br>*Only common tests should be described solely by name; describe more complex techniques in the Methods section.*
[ ]	[x]	A description of all covariates tested
[ ]	[x]	A description of any assumptions or corrections, such as tests of normality and adjustment for multiple comparisons
[ ]	[x]	A full description of the statistical parameters including central tendency (e.g. means) or other basic estimates (e.g. regression coefficient) AND variation (e.g. standard deviation) or associated estimates of uncertainty (e.g. confidence intervals)
[ ]	[x]	For null hypothesis testing, the test statistic (e.g. *F*, *t*, *r*) with confidence intervals, effect sizes, degrees of freedom and *P* value noted<br>*Give P values as exact values whenever suitable.*
[x]	[ ]	For Bayesian analysis, information on the choice of priors and Markov chain Monte Carlo settings
[ ]	[x]	For hierarchical and complex designs, identification of the appropriate level for tests and full reporting of outcomes
[ ]	[x]	Estimates of effect sizes (e.g. Cohen's *d*, Pearson's *r*), indicating how they were calculated

Our web collection on statistics for biologists contains articles on many of the points above.

Software and code

Policy information about availability of computer code

Data collection	Flow cytometry data were collected using BD FACSDiva Software V9.0 (RRID:SCR_001456)
Data analysis	Whole exome sequencing analysis<br>Exome sequencing reads were mapped to human reference genome GRCh38 using BWA V0.7.1 (RRID:SCR_010910), and duplicates were removed using Picard V1.95 (RRID:SCR_006525). The resulting BAM files were realigned around indels and recalibrated for base quality using GATK V3.5-0 (RRID:SCR_001876) with known variant sites from dbSNP-144 and the 1000 Genomes project (RRID:SCR_008801). Somatic mutations were called as tumor-normal pairs using MuTect2 (GATK V3.5-0) (RRID:SCR_000559). The SnpEFF package V4.3 (RRID:SCR_005191) was used to annotate the somatic mutations, and only variants annotated as high or moderate impact were used for downstream analysis. The Sequenza algorithm V2.1 (RRID:SCR_016662) with default parameters was used to determine the copy number profiles of bulk exome datasets. ggplot2 V3.3.3 (RRID:SCR_014601) and ComplexHeatmap V2.7.8.100 (RRID:SCR_017270) packages were used for visualization.<br>Single-cell RNA sequencing analysis<br>Raw Illumina sequencing reads were aligned to GRCh38 (human) using Cell Ranger V5 software (RRID:SCR_017344) with default parameters. Subsequently, genes were quantified as UMI counts using Cell Ranger and initially visualized using Loupe Browser V5 (RRID:SCR_018555). Downstream analysis was performed on filtered feature counts generated by Cell Ranger, and low-quality single cells containing <500 expressed genes or >20% mitochondrial transcripts or >50% ribosomal transcripts were removed. Additionally, genes expressed in fewer than three single cells were removed. We identified potential single-cell doublets using DoubletFinder V2.0.3 (RRID:SCR_018771), with an expectation of 7.5% doublet rate assuming Poisson statistics, as per the developer's code on GitHub. Following the removal of low-quality and doublet cells, single cells were normalized and clustered using Seurat V4.0.0 (RRID:SCR_016341) and batch-corrected using Harmony V1.0. Single-cell gene expression counts were normalized to the library size and log2-transformed. We applied principal component analyses (PCA) to reduce the dimensionality of the data using the top 2000 most variable genes in the dataset. Computed principal components were batch corrected for variations between patients and sex using the Harmony R package V1.0. We used batch-corrected PCs as input for Louvain-based graphing and chose resolution parameters between 0.1 and 1 depending on the single-cell datasets. Seurat V4.0.0 (RRID:SCR_016341) was used to identify cluster-specific marker genes and visualization with dot and feature plots. The genes specifically expressed in each cluster

were examined to identify the cell types. Separately, we also used reference-based R package SingleR V1.8 to identify the sub-cell types in an unbiased marker-free manner for T cells. SingleR compares expression profiles of single cells against reference transcriptomes of pure cell types to infer the cell of origin.

Classification of the tumor and normal cells (CopyKat)

All cells were classified as either normal or tumor based on the genome-wide copy number profiles computed from the gene expression UMI matrix using the Bayesian segmentation approach, CopyKat V0.1.0.

Pathway enrichment analyses

We used different approaches to identify and visualize enriched pathways in our subsets. 1. Gene Ontology Enrichment Analysis (GO). To identify enriched molecular pathways based on differentially expressed genes (DE genes), over-representation analysis (ORA) was performed on DE genes from each cluster using g:Profiler V0.2.0 (RRID:SCR_006809). Genesets from Gene Ontology (GO) biological processes, Reactome, and Kyoto Encyclopedia of Genes and Genomes (KEGG) (RRID:SCR_012773) were used. GOplot V1.0.2 was used to visualize the results. 2. Gene Set Enrichment Analysis (GSEA). We used fGSEA V1.14.0 (RRID:SCR_020938) R package to test for enrichment of the Hallmark genesets downloaded from MsigDB (RRID:SCR_016863, msigdbr R package V7.2.1). For input, we used either z-score statistics from Seurat DE analysis or pre-ranked gene lists generated using a fast Wilcoxon rank-sum test (presto R package V1.0.0 “github.com/immunogenomics/presto”). 3. Select gene set signature scoring. To generate the butterfly plots in Fig 4f, we first selected the four most significantly enriched pathways generated from GSEA analysis across our clusters. Then, we adopted the method developed by Neftel et al. to obtain single-cell scores using the “score” function from JLaffy/scrabble R package. For each gene set, a signature score (SC(i)) was calculated for each cell (i) by first quantifying the averaged relative expression of the genes in said geneset (Er) followed by normalization by subtracting the averaged relative expression of a control gene set (Ercontrol): SC(i) = Er(i) –Ercontrol(i). The control gene set was defined as described in Neftel et al. The exact position of each dot on the butterfly plot was calculated using scrabble::hierarchy() function in R using [sign(SC1-SC2)*log2(|SC1-SC2|+1)].

Comparison to in vitro-defined macrophage subtypes

To determine whether glioma-associated myeloid cells could be classified into in vitro-defined macrophage subtypes, we designed meta-modules based on known genes upregulated in M1, M2a, M2b, M2c, and M2d macrophages (Supp Fig 4f). Signature scores for each meta-module were calculated using the JLaffy/scrabble R package as above, and the results were visualized using boxplots (ggplot2 R package) (Supp Fig 4e).

Assignment of GBM subtypes

Meta-modules defined by Neftel et al. 26 were used to assign glioma molecular subtypes (MES1-like, MES2-like, NPC1-like, NPC2-like, AC-like, OPC-like) to our human. For our analyses, we collapsed the MES1 and MES2 groups into one group of MES-like cells, and similarly the NPC1 and NPC2 into one group of NPC-like cells. We used the scrabble package to calculate meta-module scores using the “score” function and develop two-dimensional plots representing cellular states, where each quadrant corresponds to one cellular state. The exact position of each dot was calculated using scrabble::hierarchy() function in R. Results were visualized using ggplot2.

Myeloid cluster signature gene validation in an independent cohort of GBMs

For the validation glioma dataset, we obtained scRNA-seq data from the Neftel et al. 10X single-cell RNA-seq dataset (#GSE131928, GEO - https://www.ncbi.nlm.nih.gov/geo/query/acc.cgi?acc=GSE131928). Count data were downloaded from GEO, and Seurat was used to generate cell clusters as described above. Cell clusters expressing myeloid cell markers were aggregated and their normalized, log-transformed expression data were used to generate the heatmaps (Supp Fig 6).

Survival prediction of glioma patients using myeloid cluster signatures

To assess the correlation between our macrophage subtype signatures and survival in glioma patients, we used publicly available datasets: the Chinese Glioma Genome Atlas (CGGA) (RRID:SCR_018802) (mRNAseq_325, Illumina HiSeq), and The Cancer Genome Atlas (TCGA) GBM dataset (RRID:SCR_003193) (http://linkedomics.org/data_download/TCGA-GBM/Human__TCGA_GBM__UNC__RNAseq__GA_RNA__01_28_2016__BI__Gene__Firehose_RSEM_log2.cct.gz)70. We analyzed the RNAseq (GA, Gene level) dataset with 528 samples to use a large dataset for our analysis. For each patient, a signature score was calculated per myeloid cluster signature genes (top 50 DE genes per cluster, DE genes were generated with Seurat::FindAllMarkers() function. Top DE genes= 50 for all clusters except MC02 which only had 14 unique DE genes). Signature scores were then generated using the “score” function from JLaffy/scrabble R package (see details above) which assigns a signature score for each cluster signature per patient. Survival analysis were done using the survival V3.2-7 (RRID:SCR_021137) and survminer V0.4.9 (RRID:SCR_021094) packages. Since signature scores are centered, patient cohorts were stratified into two groups based on the sign of the signature score (above zero= “enriched”, below zero= “not enriched”), and the statistical significance of the difference in clinical outcome was calculated using the log-rank Mantel-Cox test. The survival characteristics of the groups were visualized using Kaplan-Meier curves. Multivariate Cox regression analysis were performed using the survival::coxph() function using the variables specified in the text.

Cell-cell communication analysis using CellphoneDB

We applied an established method CellPhoneDB38 package V2.1 (RRID:SCR_017054) to study cell-cell interactions across Glioma, Myeloid and T-cell cell types. CellPhoneDB uses several ligand-receptor databases like IUPHAR, UniProt, Ensemble and PDB as a reference to evaluate the cellular communication networks between two cell types. We only considered those ligands and receptors that are expressed in at least 10% (default cutoff) of the single cells in a specific cluster. CellPhoneDB performs a pairwise comparison between all the cell types by randomly permuting labels of the clusters 1,000 times (default) and determining the mean average expression levels of ligand and receptor (LR) in the given interacting cluster pairs. Finally, CellPhoneDB computes a p-value by calculating the proportion of the means that are equal to or higher than the actual mean for a specific ligand-receptor pair. For plotting, we only considered LR pairs having p-value <= 0.05 and mean value >2 of the individual LR partner average expression in the corresponding cell type pairs.

Immunophenotyping by flow cytometry

Data were analyzed/quantified using FlowJo V10.8.0 (RRID:SCR_008520).

Statistical analysis

Statistical comparisons were performed using GraphPad Prism V9.3.0 (RRID:SCR_002798; GraphPad Software, La Jolla, CA) or R.

For manuscripts utilizing custom algorithms or software that are central to the research but not yet described in published literature, software must be made available to editors and reviewers. We strongly encourage code deposition in a community repository (e.g. GitHub). See the Nature Research guidelines for submitting code & software for further information.

Data

Policy information about availability of data

All manuscripts must include a data availability statement. This statement should provide the following information, where applicable:

- Accession codes, unique identifiers, or web links for publicly available datasets
- A list of figures that have associated raw data
- A description of any restrictions on data availability

The raw single-cell sequencing data generated in this study are publicly available with no restrictions through GEO series GSE182109 (https://www.ncbi.nlm.nih.gov/geo/query/acc.cgi?acc=GSE182109). The raw exome sequencing data generated in this study has been deposited in SRA under the accession code PRJNA787981 (https://www.ncbi.nlm.nih.gov/sra/PRJNA787981). The Neftel et al. 26 The 10X single-cell RNA-seq publicly available data used in this study is available through GEO (#GSE131928, https://www.ncbi.nlm.nih.gov/geo/query/acc.cgi?acc=GSE131928). The publicly available CGGA (Chinese Glioma Genome Atlas) dataset used in this study (mNRAseq_325) is available through the following link: http://www.cgga.org.cn/download.jsp and downloadable from http://www.cgga.org.cn/download?file=download/20200506/CGGA.mRNAseq_325.RSEM-genes.20200506.txt.zip&type=mRNAseq_325&time=20200506. The publicly available The Cancer Genome Atlas (TCGA) GBM dataset used in this study is available through
this link: vahttp://linkedomics.org/data_download/TCGA-GBM/
and downloadable using the following link: http://linkedomics.org/data_download/TCGA-GBM/Human__TCGA_GBM__UNC__RNAseq__GA_RNA__01_28_2016__BI__Gene__Firehose_RSEM_log2.cct.gz. Source data are provided with this paper. The remaining data are available within the Article, Supplementary Information or Source Data files.

Field-specific reporting

Please select the one below that is the best fit for your research. If you are not sure, read the appropriate sections before making your selection.

☒ Life sciences ☐ Behavioural & social sciences ☐ Ecological, evolutionary & environmental sciences

For a reference copy of the document with all sections, see nature.com/documents/nr-reporting-summary-flat.pdf

Life sciences study design

All studies must disclose on these points even when the disclosure is negative.

Sample size	We analyzed 44 samples from 18 glioma patients (2 low grade, 11 primary GBM and 5 recurrent GBM) patients. From these samples, we analyzed 201,986 human glioma, immune, and other stromal cells. This represents one of the biggest and most comprehensive single cell data sets for human glioma currently available. Having 201,986 single cell transcriptome dataset provided us with enough power to indentify molecular subtypes of all major cell subtypes (major findings of this paper).<br>Power analyses were used to determine appropriate sample sizes for animal experiments (power 0.8, alpha 0.05).
Data exclusions	Exclusion of cells with high mitochondrial and ribosomal RNA contents is a pre-established method for removing dead/dying cells that from confounding the analysis.<br>https://pubmed.ncbi.nlm.nih.gov/30827681/<br>“Single-Cell RNA-Seq Reveals AML Hierarchies Relevant to Disease Progression and Immunity”
Replication	This was a discovery study to identify cellular heterogeneity in human GBM. We validated our major findings (macrophage subtypes) in an independent dataset from a published study (Neftel et al) and tested survival associated with different myeloid cell subtype signatures genes in two independent human glioma datasets (CGGA and TCGA). We validated reproducibility of our functional validation studies using two independent mouse glioma models. All findings were reproducible.
Randomization	Randomization was not necessary for our single cell analysis since there were treatment groups. Our functional studies in mice also need not require randomization since there were no treatment groups. We compared B6 wildtype vs. S100a4-/- hosts and we matched age and sex of host mice and no other criteria were used for selection of host mice from our colony.
Blinding	Our single cell analysis was performed using unbiased computational methods published by others; hence, no blinding was necessary.

Reporting for specific materials, systems and methods

We require information from authors about some types of materials, experimental systems and methods used in many studies. Here, indicate whether each material, system or method listed is relevant to your study. If you are not sure if a list item applies to your research, read the appropriate section before selecting a response.

Materials & experimental systems

n/a	Involved in the study
	☒ Antibodies
	☒ Eukaryotic cell lines
☒	Palaeontology and archaeology
	☒ Animals and other organisms
	☒ Human research participants
☒	Clinical data
☒	Dual use research of concern

Methods

n/a	Involved in the study
☒	ChIP-seq
	☒ Flow cytometry
☒	MRI-based neuroimaging

Antibodies

Antibodies used

Immunophenotyping by flow cytometry
Antibodies used: PE-cy7 CD45 (#103114, RRID:AB_312979; BioLegend – 1:1000), APC-cy7 CD45 (#103116, RRID:AB_312981; BioLegend – 1:1000), BV650 CD11b (#101259, RRID:AB_2566568; BioLegend – 1:1000), PE CD3e (#100308, RRID:AB_312673; BioLegend – 1:1000), BV650 CD4 (#100469, RRID:AB_2783035; BioLegend – 1:1000), BV711 CD8a (#100747, RRID:AB_11219594; BioLegend – 1:1000), BV711 Ly6C (#128037, RRID:AB_2562630; BioLegend – 1:1000), and APC-cy7 Ly6G (#127624, RRID:AB_10640819; BioLegend – 1:1000).
Immunofluorescence analysis
Primary antibodies used: CD3 (#14-0032-85, RRID:AB_467054; Thermo Fisher Scientific -1:2000), S100A4 (#PA5-16586, RRID:AB_10977371; Thermo Fisher Scientific- 1:200), CD45 (#CBL1326, RRID:AB_2174425; MilliporeSigma, Burlington, MA- 1:200). hCD206 (#MCA2235GA, RRID: AB_322613; Bio-Rad, Hercules, CA, USA- 1:100), hS100A4(#SAB2500902, RRID: AB_10604809; MilliporeSigma, Burlington, MA- 1:100), GFP (#AHP975, RRID: AB_566990; Bio-Rad, Hercules, CA, USA- 1:200), FOXP3 (#No. 320001, RRID: AB_439745; Biolegend, San Diego, CA ,USA- 1:100), CD3(#MA1-90582, RRID: RRID:AB_1956722; Thermo Fisher scientific, Waltham, MA,USA- 1:200), CD163(#16646-1-AP, RRID: AB_2756528; Proteintech, Rosemont, IL ,USA- 1:100), mCD206(#18704-1-AP, RRID: AB_10597232; Proteintech, Rosemont, IL ,USA- 1:200) and mCD25(#No.101902, RRID: AB_312845; Biolegend, San Diego, CA ,USA- 1:200). Secondary antibodies: anti-Goat IgG Alexa Flour488 (# A-11055, RRID:AB_2534102; Thermo Fisher Scientific). Anti-Rabbit IgG Alexa Flour488 (# A-11070, RRID:AB_142134; Thermo Fisher Scientific). Anti-Rabbit IgG Alexa Flour594 (# A-11072, RRID:AB_142057;Thermo Fisher Scientific). Anti-Rat IgG Alexa Flour594 (# A-11007, RRID:AB_10561522;Thermo Fisher Scientific).
Immunohistochemistry analysis
S100A4 primary antibody (#13018 RRID:AB_2750896, Cell Signaling Tech- 1:200), anti-mouse/rabbit/goat IgG-biotinylated secondary antibody (# BA-1300, RRID:AB_2336188; Vector Laboratories)

Validation

PE-cy7 CD45 (#103114, BioLegend) https://www.biolegend.com/en-us/search-results/pe-cyanine7-anti-mouse-cd45-antibody-1903
APC-cy7 CD45 (#103116, BioLegend) https://www.biolegend.com/en-us/products/apc-cyanine7-anti-mouse-cd45-antibody-2530
BV650 CD11b (#101259, BioLegend) https://www.biolegend.com/en-us/products/brilliant-violet-650-anti-mouse-human-cd11b-antibody-7638
PE CD3e (#100308, BioLegend) https://www.biolegend.com/en-us/products/pe-anti-mouse-cd3epsilon-antibody-25
BV650 CD4 (#100469, BioLegend) https://www.biolegend.com/en-us/products/brilliant-violet-650-anti-mouse-cd4-antibody-16780
BV711 CD8a (#100747, BioLegend) https://www.biolegend.com/en-us/products/brilliant-violet-711-anti-mouse-cd8a-antibody-7926
BV711 Ly6C (#128037, BioLegend) https://www.biolegend.com/en-us/products/brilliant-violet-711-anti-mouse-ly-6c-antibody-8935
APC-cy7 Ly6G (#127624, BioLegend) https://www.biolegend.com/en-us/products/apc-cyanine7-anti-mouse-ly-6g-antibody-6755
CD3 (#14-0032-85, Thermo Fisher Scientific) This Antibody was verified by Relative expression to ensure that the antibody binds to the antigen stated. https://www.thermofisher.com/antibody/product/CD3-Antibody-clone-17A2-Monoclonal/14-0032-82
S100A4 (#PA5-16586, Thermo Fisher Scientific) https://www.thermofisher.com/antibody/product/S100A4-Antibody-Polyclonal/PA5-16586
CD45 (#CBL1326, MilliporeSigma) https://www.emdmillipore.com/US/en/product/Anti-CD45-Antibody-clone-IBL-5-25,MM_NF-05-1416
hCD206 (#MCA2235GA, Bio-Rad) https://www.bio-rad-antibodies.com/monoclonal/mouse-cd206-antibody-mr5d3-mca2235.html?f=purified
hS100A4(#SAB2500902, MilliporeSigma) https://www.sigmaaldrich.com/US/en/product/SIGMA/SAB2500902
GFP (#AHP975, Bio-Rad) https://www.bio-rad-antibodies.com/polyclonal/green-fluorescent-protein-antibody-ahp975.html?f=purified
FOXP3 (#No. 320001, Biolegend) https://www.biolegend.com/en-us/products/purified-anti-mouse-rat-human-foxp3-antibody-2886
CD3(#MA1-90582, Thermo Fisher scientific) https://www.thermofisher.com/antibody/product/CD3e-Antibody-clone-SP7-Monoclonal/MA1-90582
CD163(#16646-1-AP, Proteintech) https://www.ptglab.com/products/CD163-Antibody-16646-1-AP.htm
mCD206(#18704-1-AP, Proteintech) https://www.ptglab.com/products/MRC1-Antibody-18704-1-AP.htm
mCD25(#No.101902, Biolegend) https://www.biolegend.com/de-at/products/purified-anti-mouse-cd25-antibody-130
S100A4 primary antibody (#13018 Cell Signaling Tech) https://www.cellsignal.com/products/primary-antibodies/s100a4-d9f9d-rabbit-mab/13018

Eukaryotic cell lines

Policy information about cell lines

Cell line source(s)	We used two primary glioma tumorsphere lines that were established in our lab from spontaneous gliomas that formed in the S100ß-vERBb;p53 mouse model.
Authentication	The primary glioma tumorsphere lines used were established and genotyped in our lab from spontaneous gliomas that formed in the S100ß-vERBb;p53 mice
Mycoplasma contamination	all cell-lines tested negative for mycoplasma
Commonly misidentified lines (See ICLAC register)	no misidentified cell-lines were used

Animals and other organisms

Policy information about studies involving animals; ARRIVE guidelines recommended for reporting animal research

Laboratory animals	Freshly dissociated S100ß-vErbB;p53 tumorsphere cells were injected into the striatum of 6-8-week-old female and male C57BL6/J (IMSR Cat# JAX: 000664, RRID:IMSR_JAX:000664) or S100a4-/- (IMSR Cat# JAX:012904, RRID:IMSR_JAX:012904) syngeneic mice using a stereotaxic device (bregma: 2.8/-0.5/-3.5). Number of mice used in each experiment is indicated in figure 6,7 and supplementary figure 9. Mice were euthanized using CO2 inhalation when they displayed signs of brain tumors or experience more than 20% body weight loss, have a BCS (body condition score) of 2 or less, or they have continuous seizures or other complications associated with hindlimb paralysis. Whole brains were cut into 2mm coronal sections using a brain mold, and glioma regions were microdissected under a dissecting microscope for analysis. Mice were housed in the HMRI vivarium, which is an AAALAC accredited facility in compliance with the Guide for the Care and Use of Laboratory Animals (Protocol # AUP-0120-0003). Mice were housed in individually ventilated cages, 4 to 5 mice per cage. Room environment was maintained at 68 to 72º F (20 to 22º), 30% to 70% humidity, on a 12:12 light:dark cycle. All procedures were approved by the HMRI Animal Care and Usage Committee. B6 or S100a4-/- animals were randomly selected for this study and were age- and sex-matched at the time of the injections.
Wild animals	The study did not involve wild animals
Field-collected samples	the study did not involve field-collected samples
Ethics oversight	All procedures were approved by the HMRI Animal Care and Usage Committee.

Note that full information on the approval of the study protocol must also be provided in the manuscript.

Human research participants

Policy information about studies involving human research participants

Population characteristics	GBM is a rare cancer. Our human glioma samples were collected from 18 consenting patients, including 11 males and 7 females and 11 primary GBM, 5 recurrent GBM, and two low grade gliomas. ALL GBM samples were IDH wildtype (except for one sample for which this information is not available), represented all three molecular subtypes of GBM (Fig 2), and included both MGMT methylated and unmethylated samples (See Supplementary Table 1). Considering the difficulty in obtaining these samples, we took all comers (all consenting patients) and there was no selection upfront.
Recruitment	GBM is a rare cancer; therefore, we took all comers (all consenting patients) and there was no selection upfront on our part. As stated above, our samples were collected from a representative of GBM population. Our samples are biased towards GBM (Grade IV) samples compared to low grade gliomas. Hence, we refrained from making any conclusions regarding differences between the tumor grades.
Ethics oversight	Human tumor tissue was obtained under Institutional Review Board (IRB)-approved protocols (Pro00014547) at Houston Methodist Hospital, Houston, Texas and MD Anderson Cancer Center (PA 19-0661) in accordance with national guidelines. All patients signed informed consent during clinical visits before surgery and sample collection.

Note that full information on the approval of the study protocol must also be provided in the manuscript.

Flow Cytometry

Plots

Confirm that:

- [x] The axis labels state the marker and fluorochrome used (e.g. CD4-FITC).
- [x] The axis scales are clearly visible. Include numbers along axes only for bottom left plot of group (a 'group' is an analysis of identical markers).
- [x] All plots are contour plots with outliers or pseudocolor plots.
- [x] A numerical value for number of cells or percentage (with statistics) is provided.

Methodology

Sample preparation	Freshly dissected mouse glioma tissues were microdissected into small chunks and then treated with Accutase for 10-15 min at 37°C. Accutase was removed, and tissues were resuspended in DME/F12+B27+pen/strep medium to generate single-cell suspensions. Cells were resuspended in RBC lysis buffer to remove red blood cells. Following RBC lysis, cells were strained through 40 μm Flowmi cell strainers (#H136800040; Bel-Art, Wayne, NJ). Cells were then stained with multiple flow cytometry validated antibody cocktails
Instrument	BD Fortessa or LSRII cytometers
Software	Data were collected using BD FACSDiva Software V9.0 (RRID:SCR_001456) and analyzed/quantified using FlowJo V10.8.0 (RRID:SCR_008520).
Cell population abundance	Immune cells represented 5 to 80% of total cells in our mouse samples
Gating strategy	First, debris were excluded using FSc/SSc. Then FSc area/FSc hight were used to select singlets. Live/Dead discrimination dyes were used to select live cells and then we selected CD45+ cells for further analysis as displayed in Fig6 and supplementary Fig9. A figure exemplifying the gating strategy is provided in supplementary Fig10.

☒ Tick this box to confirm that a figure exemplifying the gating strategy is provided in the Supplementary Information.
